# Effectively engaging faith-based leaders on syringe services programs: U.S. pastors’ knowledge, perceptions, and questions

**DOI:** 10.1186/s13011-024-00620-y

**Published:** 2024-08-05

**Authors:** Betsy Smither, Philip M. Reeves, Jennifer Reynolds

**Affiliations:** Oak Ridge Associated Universities, 100 ORAU Way, Oak Ridge, TN 37831 United States of America

**Keywords:** Harm reduction, Syringe services programs, Faith-based leaders, Surveys and questionnaires, Needle-exchange programs, Clergy

## Abstract

**Objective:**

To identify faith-based leaders’ (FBLs’) knowledge, perceptions, and questions about syringe services programs (SSPs).

**Methods:**

We conducted a one-time, national online survey of 461 Christian FBLs August–September 2022.

**Results:**

56% of FBLs agreed they support having SSPs in their communities; only 7% strongly disagreed. We identified 15 main questions FBLs have about SSPs. We found statistically significant differences based on FBL Protestant affiliations. Mainline FBLs are more knowledgeable about SSPs, likely to believe a larger number of SSP services would benefit their community, supportive of SSPs, interested in data related to SSPs, and likely to look to local public health officials to shape their opinions on SSPs compared with non-mainline FBLs.

**Conclusions:**

SSP advocates can address questions that FBLs have about SSPs before beginning outreach. By understanding common Protestant denominational affiliations, advocates can focus initial engagement efforts on FBLs in their communities who are more likely to support SSPs. Our findings suggest that local public health officials can influence FBLs’ opinions about SSPs.

**Supplementary Information:**

The online version contains supplementary material available at 10.1186/s13011-024-00620-y.

## Background

According to the Centers for Disease Control and Prevention (CDC), “nearly 30 years of research has shown that comprehensive syringe services programs (SSPs) are safe, effective, and cost-saving; do not increase illegal drug use or crime; and play an important role in reducing the transmission of viral hepatitis, HIV, and other infections.” [1–3] Further, research demonstrates that SSPs protect the public and first responders from improperly disposed needles, reduce overdose deaths, and facilitate people who use drugs (PWUD) entering treatment and stopping drug use at rates far exceeding those who do not access such programs [4]. Despite the strong evidence supporting SSPs and a growing need for such programs to combat the U.S. overdose crisis, [5] advocates continue to face opposition to opening or operating SSPs in many states and communities [6]. SSP staff have “described how stigma in the community, especially lack of support from other agencies such as law enforcement, medical facilities, and public health departments” has impeded their work [7]. Harm reduction advocates are challenged to bolster community support for SSPs to ensure enough programs operate to meet the needs of PWUD throughout the United States [8]. CDC notes “partnerships and effective communication with law enforcement, elected officials, business leaders, public health, the medical community, people who inject drugs and family/friends, and the faith community can address a variety of community concerns.” [9].

Faith-based leaders (FBLs) have significant impact on the views of many community members, including politicians and other leaders who may directly influence SSP-related policies [10–12]. Research has shown that an individual’s support for punitive or protective policies towards PWUD correlates with what they expect their religious leader’s view to be on the topic, [13] and personal religiosity has not been found to be significantly associated with support for needle and syringe programs [14]. Although anecdotal reports suggest that many FBLs oppose public health interventions such as harm reduction and syringe services, [13] literature exploring FBLs’ perceptions of harm reduction strategies is sparse. Only one study was identified that explored FBLs’ perceptions on the topic. Grundy et al. (2021) surveyed rural Illinois faith leaders and found low levels of knowledge about SSPs and mixed levels of support [10].

This is the first study to assess the knowledge, attitudes, and beliefs about SSPs of a large, geographically diverse sample of U.S. Christian FBLs. It is also the first to identify FBLs’ specific questions and learning topics of interest related to SSPs, as well as to explore who FBLs cite as influencing their own opinions about SSPs.

Findings from this study can inform SSP advocates’ communication decisions with FBLs in accordance with the Diffusion of Innovation theoretical model, [15, 16] as it illuminates where FBLs currently fall within the five-step innovation-decision process.

## Methods

Oak Ridge Associated Universities (ORAU), with support from CDC, developed a 28-question survey for U.S. Christian FBLs to assess knowledge, attitudes, and beliefs in relation to SSPs, harm reduction, and substance use (Additional file S1). This article includes analysis of select survey question responses, as referenced in parentheses throughout the Results section.

We contracted Barna Group (a professional research firm) to recruit qualified respondents from their FBL panel using an approved screening questionnaire and administer the online survey. Upon completing the survey, respondents received a $25 monetary incentive as a token of appreciation.

Barna Group administered the survey from August 21–September 9, 2022, to 461 qualified FBLs. All respondents were pastors or priests working in Christian churches across the U.S. Most respondents were male (85%), over 45 years of age (68%), non-Hispanic white (93%), and considered themselves politically and socially conservative (58%). During analysis, FBLs were categorized into one of three Christian affiliations: Non-mainline Protestant (73.9%), Mainline Protestant (24.7%), and Not Protestant (1.3%). Though the difficulty in creating clear and simple distinctions among Christian identifications in social research has been documented, [11] the affiliations used for our analysis (Mainline Protestant, Non-mainline Protestant, and Not Protestant) echo similar categories used by prominent survey research institutes [17] to categorize U.S. Christians [18].

See Additional file S2 for an overview of respondent demographics. See Additional file S3 for a list of common Christian denominations, denominational groupings, and the number of survey respondents representing each as classified by Barna Group in accordance with their published Glossary of Theolographics and Demographics [19].

### Statistical analysis

The survey included both closed-ended, quantitative questions and open-ended, qualitative questions. To analyze the quantitative responses, several different analytical approaches were used based on the scale of measurement of the items. An independent t-test was conducted to examine mean differences when a continuous dependent variable was available. The Kruskal-Wallis one-way analysis of variance was used to examine median group differences on single Likert-scale items. Chi-square analyses were used to analyze categorical data. All analyses were conducted with SAS.

To analyze the qualitative responses, researchers leveraged a Grounded Theory approach, applying an open coding schema and analyzing the data using the Constant Comparative Method [20, 21]. To begin, two researchers independently reviewed 30% of the qualitative data and drafted potential primary codes for inclusion in the codebook. The researchers then met to compare codes and, after discussion, finalized a shared project codebook that was imported into NVivo software. Once coding in NVivo was complete, the two researchers again met to review and resolve any remaining coding decisions.

## Results

### SUD services provided by churches

FBLs were asked to identify any services their church provides related to SUD (Q14). They were most likely to provide referrals to community social support programs for people with SUD (64%), referrals to SUD treatment or mental health services (50%), SUD recovery meetings (34%), individual counseling with people with SUD (31%), and direct social support (e.g., housing, food, clothing) to individuals with SUD (30%). None of the respondents’ churches hosted SSPs, though 2% knew of other churches that did. 3% of FBLs distributed naloxone at their churches.

### Knowledge about SSPs and perceived need of their services

FBLs were asked to rate their level of knowledge about SSPs (Q4). The five response options ranged from “very knowledgeable” to “not at all familiar.” Only 2% of respondents were “very knowledgeable,” while a majority (58%) selected the lowest two options indicating little to no knowledge. A Kruskal-Wallis test was conducted to examine the median difference in knowledge between denominational groups. Mainline FBLs self-reported significantly higher knowledge about SSPs than non-mainline FBLs [*H* (1) = 14.12, *p* < .001].

Respondents were then asked to identify services, from a list of 10 that are commonly provided by SSPs, that would most benefit their community (Q6). The top selections were referrals to mental health services (83%) and connecting people with SUD treatment (82%). These were followed by referrals to medical services (65%) and social services (65%). Access to sterile needles, syringes, and other injection equipment was selected least often (24%). Only 4% selected that “none” of the services would benefit their community. The number of services selected was summed and a t-test was conducted to examine mean differences on the summed total, based on type of denomination. There was a statistically significant difference [*t*(164.39) = 5.25, *p* < .0001] with mainline FBLs (M = 6.37, SD = 3.08) indicating that a larger number of services offered by SSPs would benefit their communities when compared to non-mainline FBLs (M = 4.70, SD = 2.47).

### Level of support for SSPs

FBLs were then provided the following description: *SSPs are one tool communities can use to provide comprehensive services to people who inject drugs. These services include referral to substance use disorder treatment; access to sterile needles, syringes, and other injection equipment; testing for HIV and hepatitis C; education about preventing overdoses and safer injection practices; and referral to medical, mental health, and social services*(Q7). When asked their level of agreement with the statement “I support or would support having SSPs operating in my community,” more than half of FBLs agreed with 26% strongly agreeing and 30% somewhat agreeing. 20% were neutral, 17% somewhat disagreed, and 7% strongly disagreed. A Kruskal-Wallis test was conducted to examine the median difference in agreement between denominational groups. Mainline FBLs reported significantly higher support for SSPs operating in their community than non-mainline FBLs [*H* (1) = 56.82, *p* < .001].

When asked “how open or willing are you to change your opinion,” 60% of FBLs indicated that they were very open to the possibility, 30% were somewhat open, and only 10% were not at all open to the possibility of changing their opinions or perspectives of SSPs (Q8). A Kruskal-Wallis test was conducted to examine the median difference in willingness to change between denominational groups. There was not a statistically significant difference between mainline and non-mainline FBLs [*H*(1) = 0.29, *p* = .59].

A Spearman-Rank Order correlation was conducted between Q7 and Q8 to test the association between variables. For all participants, the correlation was statistically significant *r*(459) = 0.257, *p* < .001. The correlation was weak but suggested that less support for SSP’s operating in their community is related to less openness to changing their opinion. Among those who strongly disagreed with supporting SSPs in their community, none were “very open” to changing their opinion.

Respondents were asked in an open-ended question, “If you were approached by an organization interested in opening an SSP in your community and who asked for support, how would you respond?” (Q20). Researchers identified 10 qualitative codes that overview FBLs’ responses. These ranged from full and immediate support (*n* = 73, 16%) to unequivocal, ideological disagreement with SSPs (*n* = 54, 12%). A majority of FBLs offered more nuanced responses that fell along a continuum of support. The most frequent responses, (*n* = 182, 39%) were coded under “Openly ask questions/request information.” Codes and the number of references in each are shown in descending order from most to least supportive (as characterized by the researchers) (Table 1).

**Table 1:** Anticipated responses to requests to support community SSPs (Q20)

Codes	# of References	Verbatim Example
Offer unqualified, immediate support	*n* = 73, 16%	*I would personally support it and be willing to help with educational opportunities for my congregation.*
Offer spiritual supports (e.g., teaching faith-based classes, pastoral counseling)	*n* = 3, 1%	*I would be open as long as spiritual issues could be addressed. Jesus can change lives.*
Talk to others in church about it (e.g., existing counsels, boards)	*n* = 66, 14%	*I would schedule a time to meet bringing with me another staff or volunteer leader. After hearing the range of ways we could be supportive*, *bring those back to our congregational leadership for prayer and evaluation.*
Openly ask questions/request information	*n* = 182, 39%	*I would want a lot of information since I know very little about the subject. I would also talk to a friend who works in public health about it.*
Only refer people to the SSP (no other involvement or collaboration)	*n* = 3, 1%	*I would probably decline to host or to give vocal support as my church leadership would not be on board with it. However*, *I would refer people to some of the services of an SSP.*
Not sure or neutral	*n* = 31, 7%	*I would not know how to respond. I don’t know anything about SSPs.*
Decline to provide tangible resources (e.g., money, space)	*n* = 11, 2%	*Our church is not equipped to host the SSP*, *and our staff does not have the discretionary time to participate.*
Be willing to cautiously listen	*n* = 46, 10%	*Tell me more about your program and why it would be a benefit rather than simply an opportunity for codependency?*
Refer the SSP organization to other community-based organizations	*n* = 5, 1%	*I would encourage them to seek secular support and I would be willing to work with law enforcement as a Police chaplain. I am not comfortable with the idea of my church supplying drug paraphernalia.*
Decline support on ideological grounds	*n* = 54, 12%	*SSPs might be useful in a secular setting but in a church setting we cannot participate in perpetuating the sin and destruction of illicit drug use and so we cannot be involved in or host SSPs*

### Information needs

Next, respondents were asked, “If an SSP was opening in your city, what questions would you want answered?” (Q21). Researchers coded the 667 questions (some respondents provided multiple questions) into 15 main categories shown in descending order by frequency (Table 2).

**Table 2:** Questions about SSPs (Q21)

Question Categories	*N*=	% of 461
Do SSPs really help in the long run; *don’t they just enable drug use*? What proportion of clients enter long-term recovery and experience “life-change,” “deliverance,” or “freedom from addiction”? Do most clients simply continue drug use, but just in a safer manner?	131	20%
What impact do SSPs have on *community safety and crime rates*? Will an SSP draw more people who use drugs to our city? Will an SSP have negative impacts on the surrounding community?	82	18%
What specific *services* will be offered?	72	16%
Please describe the *operations and staffing* at this SSP (e.g., policies, procedures, staff roles and qualifications).	70	15%
What is the main *mission*, *visions*, *goals*, *and philosophies* that will underpin this SSP?	50	11%
Please describe the *funding*, *budget*, *and expected economic impact* of the SSP.	45	10%
Where, specifically, will it be *located*?	41	9%
How will clients *access services*? What are the referral and screening processes?	37	8%
*How can churches support* this SSP?	35	8%
Does the SSP have any plans for addressing the *spiritual health of clients*? Is the SSP open to offering services from Christian viewpoints (e.g., pastoral care, chaplains)?	23	5%
What are the demonstrated *impacts*, *success rates*, *or effectiveness data* for SSPs?	21	5%
What *community partners* are supporting this effort, and how will the SSP integrate with the other organizations already working with people with SUD locally?	19	4%
How will you communicate *basic education about SSPs and/or raise awareness* about them in our community?	13	3%
What is the *demonstrated need* for an SSP here?	12	3%
What has been the *experience of other*, *similar cities* that have SSPs?	12	3%
What processes will be in place to prevent additional *needle litter*?	4	1%

Respondents were then asked to select up to three topics that they were most interested in learning more about from a list of 16 (Q23). The five most frequently selected topics were: statistics on how many SSP clients enter long-term recovery and engage productively in their communities (58%); testimonies of Christians in recovery who have used SSPs (41%); whether SSPs save lives (28%); whether SSPs provide referral to medical, mental health, and social services (26%); and testimonies from other church leaders who support SSPs (24%).

Chi-square tests were conducted to examine if the proportion of FBLs who were interested in learning about select topics significantly differed based on denomination type (Table 3). Mainline FBLs selected “whether SSPs save lives” [*X*^2^(1, *N* = 455) = 10.44, *p* = .001], “whether SSPs provide referrals to medical, mental health, and social services” [*X*^2^(1, *N* = 455) = 7.29, *p* = .007], and “statistics on how many SSP clients enter long-term recovery and engage productively in their communities” [*X*^2^(1, *N* = 455) = 12.85, *p* < .001] at a higher-than-expected rate (when compared with an equal distribution). Non-mainline FBLs selected “testimonies from other church leaders who support SSPs” [*X*^2^(1, *N* = 455) = 8.12, *p* = .004] and “testimonies of Christians in recovery who have used SSPs” at a higher-than-expected rate [*X*^2^(1, *N* = 455) = 8.93, *p* = .003].

**Table 3:** Actual and expected frequencies of mainline and non-mainline FBLs’ interest in select topics

			Long-term Recovery Rates of SSP Clients	Testimonies from Christians in Recovery	Lives Saved	Whether SSPs Make Referrals	Testimonies from FBLs who Support SSPs
Mainline	Actual	N	83.00	33.00	46.00	73.00	16.00
	Expected	N	66.15	47.10	32.07	29.56	27.81
Non-mainline	Actual	N	181.00	155.00	82.00	77.00	95.00
	Expected	N	197.85	140.89	95.93	88.44	83.19
Total	Actual	N	264.00	188.00	128.00	118.00	111.00
		%	58.02%	41.32%	28.13%	25.93%	24.40%

### Sources who influence FBLs’ opinions about SSPs

Respondents were asked who would influence their opinions about SSPs (Q9). They selected up to three options from a list of eleven. Local public health officials were the most selected source (57%), followed by other local faith-based leaders (42%), and local law enforcement (42%). 28% said they would look to state or federal health officials. Very few said they would look to national (3%) or local news (2%).

Chi-square tests were conducted to examine if the proportion of FBLs who selected each source differed based on denomination type (Table 4). Mainline FBLs selected local public health officials (*X*^2^(1, *N* = 455) = 30.73, *p* < .001), local education leaders (*X*^2^(1, *N* = 455) = 9.75, *p* = .001), and state or federal health officials (*X*^2^(1, *N* = 455) = 19.04, *p* < .001) at a higher-than-expected rate. Non-mainline FBLs selected other religious leaders in their community at a higher-than-expected rate (*X*^2^(1, *N* = 455) = 18.89, *p* < .001).

**Table 4:** Actual and expected frequencies of sources that influence mainline and non-mainline FBLs’ opinions about SSPs

			Local Public Health Officials	Local Education Leaders	State or Federal Health Officials	Other Local Religious Leaders
Mainline	Actual	N	91.00	30.00	51.00	28.00
	Expected	N	65.12	18.79	32.32	48.36
Non-mainline	Actual	N	169.00	45.00	78.00	165.00
	Expected	N	194.86	56.21	96.68	144.64
Total	Actual	N	260.00	75.00	129.00	193.00
		%	57.14%	16.48%	28.35%	42.42%

## Increasing support for SSPs among FBLs

In a final open-ended question, respondents were asked, “What, if anything, do you think could increase support for SSPs among church leaders like yourself?” (Q22). The 506 unique responses (some respondents provided multiple suggestions) were coded into three main codes: more information (48%), a productive dialogue between SSP advocates/public health and FBLs (23%) and demonstrating a strong connection to treatment/recovery for clients (16%). 9% of responses were coded to “nothing could increase my support” and 7% to “not sure what would increase support.” There was a strong overlap between the types of information FBLs indicated would increase their support for SSPs and the questions they wanted answered in Table 2.

FBLs gave many specific suggestions for the types of dialogues between FBLs and SSP advocates that could increase FBL support. These included conversations that illuminate where faith-based organizations and SSP missions may overlap, such as if the SSP will incorporate holistic health approaches to include spiritual health (11%). Some respondents (8%) also wanted to hear personal testimonies, primarily from individuals in recovery who had used SSPs previously, but also from clinicians or other FBLs who support SSPs. Others suggested that advocates facilitate an SSP focused dialogue among local FBLs (2%) on the topic or demonstrate curiosity about or respect for what FBLs are already doing regarding SUD in the community (1%).

### Limitations

Our study was subject to limitations. Our sample did not include FBLs in religions other than Christianity. We elected to focus our initial research with Christian FBLs considering 92% of Americans who are religiously affiliated identify as Christian [22]. Our respondents were primarily white (93%) and male (91%) thus do not fully represent the roughly 13% of pastors who are women nor the 27% of pastors who are Black or African American (11.7%), Hispanic or Latino (9.0%), or Asian (6.6%) [23]. Our sample did not include enough non-Protestant Christian FBLs (i.e., Catholic and Orthodox FBLs) to conduct a meaningful standalone analysis of their views. Further research is needed to explore opinions of SSPs among a more diverse sample of U.S. FBLs.

## Discussion

### FBL openness to SSPs

Public health and harm reduction advocates have previously described religious individuals, particularly religious conservatives, as opposed to harm reduction [24, 25]. Yet, our findings from a survey of predominately older, white, male, and conservative U.S. Christian FBLs suggest many are open to taking a supportive view of SSPs.

Our respondents had a low level of baseline knowledge about SSPs, which is consistent with a study of rural FBLs in the Illinois Delta Region [10]. After reading a description of SSP services that included distribution of needles, more than half of FBLs said they support SSPs operating in their communities (again similar to the Illinois Delta Region study [10]). Only 7% of our sample strongly disagreed with support for SSPs. 90% of respondents indicated openness (30% very open, 60% somewhat open) to changing their opinions about SSPs. When we qualitatively explored what their responses would be if asked to support a new SSP in their community, less than a third of respondents offered a definitive response to indicate if they would support or decline to support a new SSP. Rather, most FBLs’ responses outlined additional actions they would consider before determining their church’s level of support for a new SSP. Taken together, these findings signal that many FBLs may have nascent views on SSPs that can be shaped by additional information.

### Communicating with FBLs about SSPs

Public health officials are well positioned to answer FBLs’ questions (Table 2) and can be encouraged that they were cited as the most frequent source FBLs would look to when shaping their opinions about SSPs. Notably, each of the top three sources FBLs stated they would look to when shaping their opinions on SSPs were local (i.e., local public health, other local faith-based leaders, and local law enforcement), which indicates the importance of community-based conversations around SSPs.

FBLs expressed interest in hearing answers to a broad array of questions about SSPs. FBLs’ top question centered on if SSPs “really help in the long run” or “just enable drug use.” They were most interested to learn how many SSP clients enter long-term recovery and engage productively in their communities. They were also interested in hearing testimonies of Christians in recovery who have used SSPs. This indicates there is a strong interest among many FBLs in understanding the degree to which SSP clients enter recovery versus continuing drug use in a safer manner. While entry to treatment is not the primary goal of harm reduction, [26] it may be important for SSP advocates to be prepared to address this concern when engaging with FBLs who are still forming their opinions about SSPs. Among FBLs’ less frequent questions, only 5% wanted to know if SSPs address spiritual health or incorporate Christian teachings. Of note, only 1% asked a question about needle litter, which counters studies on other stakeholder groups that suggest needle litter is a primary concern [27].

To summarize, our survey findings suggest creating education materials for FBLs that:

- Assume a low baseline level of knowledge about SSPs but an openness to forming a supportive stance towards them.
- Promote offered SSP services that meet FBLs’ perceived needs for the community (provision of or referrals to SUD treatment; mental health, medical, and social services).
- Provide answers to Table 2 questions that draw on national data, [28] findings from SSP case studies in similar communities, endorsements from key local stakeholders (i.e., public health officials, FBLs, law enforcement leaders), and plans specific to the operations of the local SSP.
- Feature testimonials (e.g., compelling quotes, photos, video statements) from Christians in recovery who have used SSPs and/or other FBLs who support SSPs.

These materials can be shared as part of a “productive dialogue between SSP advocates/public health and FBLs” in which shared goals for the community are discussed, FBLs’ viewpoints are heard respectfully, and the work FBLs are already doing with people who have SUD is acknowledged.

### Importance of understanding FBLs’ denominational affiliations

We tested for statistical significance on a variety of demographic factors and found the greatest number of differences based on FBLs’ affiliations. Understanding Mainline Protestant, Non-mainline Protestant, and Not Protestant affiliations can assist harm reduction advocates in identifying the FBLs in their community who are most likely to support SSP efforts. Our results show that, when compared with non-mainline protestant FBLs, mainline FBLs (a) self-report being more knowledgeable about SSP services, (b) believe a larger number of services commonly offered by SSPs would benefit their community, (c) are more supportive of SSPs operating in their community, (d) indicate more interest in public health data and statistics related to SSPs, and (e) are more likely to look to local public health officials to shape their opinions on SSPs. Conversely, non-mainline FBLs were more likely to say they would look to other FBLs in their community to shape their own opinions about SSPs and expressed more interest in hearing testimonies from Christians in recovery who have used SSPs. This could indicate an opportunity for public health and harm reduction advocates to focus their initial outreach efforts on mainline FBLs. Once advocates have earned the support of mainline FBLs, those FBLs could then potentially conduct outreach with their non-mainline peers.

## Conclusions

FBLs have significant influence in many communities and can be important stakeholders to engage when seeking to bolster support for local SSPs. Our findings suggest that local public health officials can influence FBLs’ opinions about SSPs in their communities. By taking the time to understand common U.S. Protestant mainline and non-mainline denominational affiliations (Additional file S3), harm reduction advocates may be able to identify the mainline FBLs in their community with whom they may want to focus their initial outreach efforts. These FBLs may also be more effective conduits for subsequent outreach to their non-mainline peers in the community. Harm reduction advocates can also prepare for outreach to FBLs by proactively organizing responses to common questions FBLs have (Table 2) about SSPs before meeting with them.

### Electronic supplementary material

Below is the link to the electronic supplementary material.

Supplementary Material 1

Supplementary Material 2

Supplementary Material 3

## Data Availability

The datasets used during the current study are available from the corresponding author on reasonable request.

## Abbreviations

CDC: Centers for Disease Control and Prevention
FBL: Faith-based leader
PWUD: People who use drugs
SUD: Substance use disorders
SSP: Syringe services program
