# Supplementary material for: Effectively engaging faith-based leaders on syringe services programs: U.S. pastors’ knowledge, perceptions, and questions
Source: Subst Abuse Treat Prev Policy. 2024 Aug 5;19:37. doi: 10.1186/s13011-024-00620-y (PMC11302828; doi:10.1186/s13011-024-00620-y)
Supplement: Supplementary file 1 — Supplementary Material 1 [file 13011_2024_620_MOESM1_ESM.docx]

# Authors/Institutions:

Betsy Smither^1^ (<betsy.smither@orau.org>), Philip M. Reeves^1^ (phil.reeves@orau.org), and Jennifer Reynolds^1^ (jennifer.reynolds@orau.org)

1. Oak Ridge Associated Universities, 100 ORAU Way, Oak Ridge, TN 37831, United States of America

|  | **Total** | |
| --- | --- | --- |
|  | **N** | **%** |
| **Gender** | | |
| Male | 421 | 91% |
| Female | 37 | 8% |
| Other | 0 | 0% |
| Prefer not to answer | 3 | 1% |
| **Age** | | |
| Under 45 | 150 | 32% |
| 45+ | 311 | 68% |
| **Years of Professional Experience** | | |
| 1–9 years | 16 | 3% |
| 10–19 years | 163 | 35% |
| 20+ years | 282 | 61% |
| **Race** | | |
| White | 427 | 93% |
| Black/African American | 8 | 2% |
| Asian/Asian American | 5 | 1% |
| Other | 9 | 2% |
| Prefer not to answer | 9 | 2% |
| **Of Hispanic, Latino, or Spanish Origin** | | |
| Yes | 12 | 3% |
| No | 449 | 97% |
| **Geographic Region** | | |
| Northeast | 65 | 14% |
| Midwest | 115 | 25% |
| South | 221 | 48% |
| West | 60 | 13% |
| **Community Type** | | |
| Urban | 57 | 12% |
| Suburban | 188 | 41% |
| Small Town | 161 | 35% |
| Rural | 56 | 12% |
| **Church Attendance** | | |
| Small (<100) | 207 | 45% |
| Medium (100–250) | 175 | 38% |
| Large (>250) | 79 | 17% |
| **Political/Social Leanings** | | |
| Mostly conservative | 267 | 58% |
| Mostly liberal | 81 | 18% |
| Somewhat in-between | 104 | 23% |
| Other | 9 | 2% |
| **Total Participants** | **461** | **100%** |
|  |  |  |
