# Supplementary material for: Effectively engaging faith-based leaders on syringe services programs: U.S. pastors’ knowledge, perceptions, and questions
Source: Subst Abuse Treat Prev Policy. 2024 Aug 5;19:37. doi: 10.1186/s13011-024-00620-y (PMC11302828; doi:10.1186/s13011-024-00620-y)
Supplement: Supplementary file 2 — Supplementary Material 2 [file 13011_2024_620_MOESM2_ESM.docx]

# Authors/Institutions:

Betsy Smither^1^ (<betsy.smither@orau.org>), Philip M. Reeves^1^ (phil.reeves@orau.org), and Jennifer Reynolds^1^ (jennifer.reynolds@orau.org)

1. Oak Ridge Associated Universities, 100 ORAU Way, Oak Ridge, TN 37831, United States of America
2. Once someone has an opioid addiction/opioid use disorder there is not much they can do about it.
   1. Strongly agree
   2. Agree
   3. Neither agree nor disagree
   4. Disagree
   5. Strongly disagree
3. If I found out that a close friend had an opioid addiction/opioid use disorder, I would avoid him or her.
   1. Strongly agree
   2. Agree
   3. Neither agree nor disagree
   4. Disagree
   5. Strongly disagree
4. If I had an opioid addiction/opioid use disorder, I would not tell anyone.
   1. Strongly agree
   2. Agree
   3. Neither agree nor disagree
   4. Disagree
   5. Strongly disagree
5. Which of the following best describes your current level of knowledge about syringe services programs (SSPs). *These are sometimes referred to as “needle exchange programs.”*

*Please select one.*

[randomize]

- 1. I am very knowledgeable about SSPs
  2. I am somewhat familiar with SSPs
  3. I have a basic understanding of SSPs
  4. I have heard of SSPs, but know little about them
  5. I am not at all familiar with SSPs

1. Which of the following services do you know or understand SSPs may provide?

*Please select all that apply.*

[randomize]

- 1. Access to sterile needles, syringes, and other injection equipment
  2. Safe disposal of used injection equipment
  3. Naloxone (a medication that reverses the effects of an overdose)
  4. Testing for HIV
  5. Testing for Hepatitis C
  6. Connecting people with substance use disorder treatment (drug treatment)
  7. Referral to medical services
  8. Referral to mental health services
  9. Referral to social services
  10. Routine adult vaccines
  11. I wasn’t aware SSPs provided any of the above [**exclusive**][**anchor**]

1. Of the following, which do you feel your community could benefit the most from having (or from having more of)?

*Please select all that apply.*

[randomize]

- 1. Access to sterile needles, syringes, and other injection equipment
  2. Safe disposal of used injection equipment
  3. Naloxone (a medication that reverses the effects of an overdose)
  4. Testing for HIV
  5. Testing for Hepatitis C
  6. Connecting people with substance use disorder treatment (drug treatment)
  7. Referral to medical services
  8. Referral to mental health services
  9. Referral to social services
  10. Routine adult vaccines
  11. None of the above [**exclusive**][**anchor**]

**Syringe services programs (SSPs) are one tool communities can use to provide comprehensive services to people who inject drugs. These services include referral to substance use disorder treatment, access to sterile needles, syringes, and other injection equipment testing for HIV and hepatitis C, education about preventing overdoses and safer injection practices, and referral to medical, mental health, and social services.**

1. How much do you agree or disagree with the following statements? “I support or would support having SSPs operating in my community?
   1. Strongly agree
   2. Somewhat agree
   3. Neutral
   4. Somewhat disagree
   5. Strongly disagree
2. When it comes to your current opinions or perspectives of SSPs, how open or willing are you to change your opinion?
   1. Very open to the possibility of changing my opinion
   2. Somewhat open to the possibility of changing my opinion
   3. Not at all open to the possibility of changing my opinion
3. Who would you be most likely to look to help shape your own opinions about SSPs?

*Please select up to three.*

- 1. Local law enforcement leaders
  2. Local government leaders (mayor, city council, etc.)
  3. Local public health officials
  4. Local education leaders
  5. State or federal government leaders
  6. State or federal health officials
  7. Other religious leaders in my community
  8. Other leaders in my denomination
  9. Local news
  10. National news or media
  11. Other (Please specify) [**open response**]
  12. I can’t think of any individual or organization that would influence my position on SSPs.

1. Now, thinking about your denomination, which do you feel best reflects your denomination’s opinion on SSPs? “Overall, my denomination is…’
   1. Strongly in support of SSPs
   2. Somewhat in support of SSPs
   3. Neutral toward SSPs
   4. Somewhat in opposition of SSPs
   5. Strongly opposed to SSPs
   6. Has not considered SSPs
   7. I’m not sure of my denomination’s position on SSPs
2. [IF PREV=1,2,3,4,5] When it comes to your denomination’s position on SSPs, how willing or open to change do you feel your denomination is?
   1. Very open to change
   2. Somewhat open to change
   3. Not at all open to change
   4. I’m not sure
3. Now thinking about your own church, which do you feel best reflects your congregation’s position on SSPs. “My congregation is…”
   1. Strongly in support of SSPs
   2. Somewhat in support of SSPs
   3. Neutral toward SSPs
   4. Somewhat in opposition of SSPs
   5. Strongly opposed to SSPs
   6. Has not considered SSPs
   7. I’m not sure of my congregation's position on SSPs
4. [IF PREV=1,2,3,4,5] When it comes to your congregation's position on SSPs, how willing or open to change do you feel your congregants are?
   1. Very open to change
   2. Somewhat open to change
   3. Not at all open to change
   4. I’m not sure
5. Of the following services, which, if any, does your church provide?

*Please select all that apply.*

[randomize]

- 1. Recovery meeting hosting (Alcoholics Anonymous, Celebrate Recovery, Al-Anon, etc.)
  2. One-on-one counseling with individuals with substance use disorders
  3. Referrals to substance use treatment or mental health services
  4. Referrals to community social support programs (e.g., housing, food, clothing) to individuals dealing with substance use disorders
  5. Direct social support (e.g., housing, food, clothing) to individuals dealing with substance use disorders
  6. Distribution of naloxone or Narcan
  7. Syringe services program hosting
  8. Teaching on substance use disorders from the pulpit
  9. Educational opportunities about substance use disorders in small group settings
  10. Financial support of treatment or recovery organizations in your community
  11. Other (Please specify) [**open response**]

1. [IF PREV=1] What recovery meetings does your church provide?

[**open response**]

1. Of the following services, which, if any, does your denomination provide?
   1. Recovery meeting hosting (Alcoholics Anonymous, Celebrate Recovery, Al-Anon, etc.)
   2. One-on-one counseling with individuals with substance use disorders
   3. Referrals to substance use treatment or mental health services
   4. Referrals to community social support programs (e.g., housing, food, clothing) to individuals dealing with substance use disorders
   5. Direct social support (e.g., housing, food, clothing) to individuals dealing with substance use disorders
   6. Distribution of naloxone or Narcan
   7. Syringe services program hosting
   8. Teaching on substance use disorders from the pulpit
   9. Educational opportunities about substance use disorders in small group settings
   10. Financial support of treatment or recovery organizations in your community
   11. Other (Please specify) [**open response**]
2. How much impact would you say that substance use disorders have on the following groups?

[grid options]

- 1. Very significant impact
  2. Moderate impact
  3. Little impact
  4. No impact at all
  5. [grid statements][randomize]
     1. Myself
     2. My family
     3. My church
     4. My local community
     5. People in my city
     6. People in my state

1. Thinking about all the social, community concerns on which you would like your church to engage, where does substance use fall in comparison with other social concerns (e.g., alleviating poverty, mentoring at-risk youth)?
   1. Very high priority
   2. Moderate priority
   3. Low priority
   4. Not at all a priority
2. Currently, what organizations in your community would you direct someone to if they had a substance use disorder?

[**open response**]

1. If you were approached by an organization interested in opening an SSP in your community and who asked for support, how would you respond?

[**open response**]

1. If an SSP was opening in your city, what questions would you want answered?

[**open response**]

1. What, if anything, do you think could increase support for SSPs among church leaders like yourself?

[**open response**]

1. Of the following topics, which are you most interested in learning more about?

*Please select up to 3.*

[randomize]

- 1. Whether SSPs reduce the risk of needle-stick injuries for law enforcement personnel
  2. Whether SSPs reduce used needle litter in communities
  3. Whether SSPs are associated with increased crime rates
  4. The legality of SSPs
  5. Whether SSPs reduce HIV rates
  6. Whether SSPs reduce hepatitis C rates
  7. Whether SSPs save lives
  8. Whether SSPs provide referrals to medical, mental health, and social services
  9. Whether SSPs provide birth control for people who use drugs
  10. Respected commentary on scriptural interpretations related to substance use disorders
  11. Testimonies from other church leaders who support SSPs
  12. Testimonies of Christians in recovery who have used SSPs
  13. SSP endorsements from Christian denominations
  14. My own denominational leaders’ views on SSPs
  15. Statistics on how many SSP clients enter long-term recovery and engage productively in their communities.
  16. None of these are compelling or interesting to me [**exclusive**][**anchor**]

1. Of the following, how do you think each would affect support for SSPs among church leaders?

[grid options]

- 1. Very likely to increase support
  2. Somewhat likely to increase support
  3. No impact on support
  4. Somewhat likely to decrease support
  5. Very likely to decrease support

[grid statements][randomize]

1. As a pastor, of the following, which messages are more appealing to you?

[randomize] [2 point semantic scale]

1. Please select any of the following continuing education or professional development opportunities in which you routinely participate.
   1. Formal seminary or other theological training courses
   2. Publications from my denomination (e.g., newsletters)
   3. Podcasts
   4. Blogs
   5. Regular meetings with other religious leaders in my community
   6. Regular meetings with other church leaders in my denomination
   7. In-person conferences or seminars
   8. Webinars or online conferences
   9. Books
2. Please list specific sources that you most frequently use to stay current in your field of up-to-date on what is happening in your denomination.

[**open response**]

1. Currently, how often do you check, visit, or listen to each of the following?

[grid options]

- 1. Throughout the day
  2. At least once a day
  3. Weekly
  4. Monthly or less
  5. Never

[grid statements][randomize]

- - 1. Facebook
    2. Instagram
    3. YouTube
    4. Twitter
    5. TikTok
    6. Snapchat
    7. Reddit
    8. Local newspapers
    9. Local television
    10. National newspapers
    11. National television
    12. Radio
    13. Podcasts
