# Supplementary material for: Effectively engaging faith-based leaders on syringe services programs: U.S. pastors’ knowledge, perceptions, and questions
Source: Subst Abuse Treat Prev Policy. 2024 Aug 5;19:37. doi: 10.1186/s13011-024-00620-y (PMC11302828; doi:10.1186/s13011-024-00620-y)
Supplement: Supplementary file 3 — Supplementary Material 3 [file 13011_2024_620_MOESM3_ESM.docx]

# Authors/Institutions:

Betsy Smither^1^ (<betsy.smither@orau.org>), Philip M. Reeves^1^ (phil.reeves@orau.org), and Jennifer Reynolds^1^ (jennifer.reynolds@orau.org)

1. Oak Ridge Associated Universities, 100 ORAU Way, Oak Ridge, TN 37831, United States of America

| **Identification** | **Category** | **# of Respondents** |
| --- | --- | --- |
| Adventist – Seventh-day or other | Non-mainline | 7 |
| AME/African Methodist | Mainline | 0 |
| AME Zion | Non-mainline | 1 |
| Anglican | Non-mainline | 3 |
| Assembly of God | Non-mainline | 14 |
| Baptist – American | Mainline | 5 |
| Baptist – Missionary | Non-mainline | 1 |
| Baptist – National | Non-mainline | 1 |
| Baptist – Southern | Non-mainline | 64 |
| Baptist – other | Non-mainline | 45 |
| Bible Church | Non-mainline | 7 |
| Brethren | Non-mainline | 2 |
| Catholic / Roman Catholic | Not Protestant | 6 |
| Christian & Missionary Alliance | Non-mainline | 8 |
| Christian/Disciples of Christ | Non-mainline | 4 |
| Christian Church / Church of Christ | Non-mainline | 15 |
| Church of Christ – independent | Non-mainline | 12 |
| United Church of Christ | Mainline | 8 |
| Church of God in Christ (COGIC) | Non-mainline | 0 |
| Church of God – Anderson | Non-mainline | 2 |
| Church of God – other | Non-mainline | 3 |
| Episcopal | Mainline | 9 |
| Evangelical Covenant / Free | Non-mainline | 5 |
| Foursquare | Non-mainline | 8 |
| Independent Fundamentalist | Non-mainline | 0 |
| Lutheran – Evangelical (ELCA) | Mainline | 17 |
| Lutheran – Missouri Synod (LCMS) | Non-mainline | 33 |
| Lutheran – WELS | Non-mainline | 6 |
| Mennonite | Non-mainline | 1 |
| Methodist - Free | Non-mainline | 6 |
| Methodist – United | Mainline | 50 |
| Methodist – other | Non-mainline | 1 |
| Nazarene | Non-mainline | 7 |
| Non-denominational / community / independent | Non-mainline | 49 |
| Orthodox / Greek Orthodox | Not Protestant | 0 |
| Pentecostal / Foursquare | Non-mainline | 2 |
| Pentecostal – United | Non-mainline | 0 |
| Presbyterian Church in America – PCA | Non-mainline | 3 |
| Presbyterian Church USA – PCUSA | Mainline | 25 |
| Presbyterian Church - Evangelical | Non-mainline | 0 |
| Reformed – Other | Non-mainline | 7 |
| Wesleyan | Non-mainline | 9 |
| Vineyard | Non-mainline | 0 |
| Other (Please specify) [open response] | Non-mainline | 15 |
| **Total Number of Respondents** |  | **461** |
